# Fluoroquinolone resistance among Streptococcus pneumoniae in Hong Kong linked to the Spanish 23F clone.

**DOI:** 10.3201/eid0705.010526

**Published:** 2001

**Authors:** P L Ho, W C Yam, T K Cheung, W W Ng, T L Que, D N Tsang, T K Ng, W H Seto

**Affiliations:** University of Hong Kong Special Administrative Region, China. plho@hkucc.hku.hk

## Abstract

Serotypes 6A/B, 19F, and 23F accounted for 73% of 140 mucosal isolates of Streptococcus pneumoniae from Hong Kong. In pulsed-field gel electrophoresis analysis, a group of related patterns was shared by 14 of 15 ciprofloxacin-resistant and 12 of 16 ciprofloxacin-susceptible isolates. These strains exhibited capsular switching and were highly similar to the Spanish 23F clone.


### Fluoroquinolone Resistance among *Streptococcus pneumoniae* in Hong Kong Linked to the Spanish 23F Clone

**Pak Leung Ho,* Wing Cheong Yam,* Terence K.M. Cheung,* Wilson W.S. Ng,* Tak Lun Que,† Dominic N.C. Tsang,‡ Tak Keung Ng,§ and Wing Hong Seto***
University of Hong Kong, Hong Kong Special Administrative Region (SAR), China; †Tuen Mun Hospital, Hong Kong SAR, China; ‡Queen Elizabeth Hospital, Hong Kong SAR, China; and §Princess Margaret Hospital, Hong Kong SAR, China

Serotypes 6A/B, 19F, and 23F accounted for 73% of 140 mucosal isolates of *Streptococcus pneumoniae* from Hong Kong. In pulsed-field gel electrophoresis analysis, a group of related patterns was shared by 14 of 15 ciprofloxacin-resistant and 12 of 16 ciprofloxacin-susceptible isolates. These strains exhibited capsular switching and were highly similar to the Spanish 23F clone.

*Streptococcus pneumoniae*, the most important cause of community-acquired pneumonia worldwide, particularly affects young children, elderly persons with chronic cardiopulmonary conditions, and immunosuppressed patients of all ages. Widespread emergence of antimicrobial resistance has become a concern in recent years. In many countries, rates of resistance to penicillin are >40%. Among penicillin-resistant *S. pneumoniae*, 60% to 90% are also resistant to the macrolides, tetracyclines, chloramphenicol, clindamycin, and cotrimoxazole. For this reason, newer fluoroquinolones with expanded activity against gram-positive bacteria have been recommended by the Infectious Disease Society of America as initial treatment of choice for community-acquired pneumonia (1).

Although resistance to the newer fluoroquinolones remains rare in most countries, the percentage of nonsusceptible *S. pneumoniae* has increased from <0.5% for ofloxacin to 5.5% for levofloxacin (MIC $\geq$4 µg/mL) from 1995 to 1998 in Hong Kong (2). Almost all strains of fluoroquinoline-resistant *S. pneumoniae* were isolated from respiratory tract specimens. Knowledge of the serotype distribution of *S. pneumoniae*, particularly strains with the emerging resistance pattern, is important for development of conjugate vaccines.

We studied the serotype distribution of recent isolates of drug-resistant pneumococci from Hong Kong, including isolates with resistance to the fluoroquinolones. To understand better the emergence of fluoroquinolone-resistant *S. pneumoniae* in this locality, we used pulsed-field gel electrophoresis (PFGE) to compare strains.

### The Study

We examined sputum isolates of *S. pneumoniae* obtained from four regional laboratories in Hong Kong during a prospective regional survey in 1998 (2). The four laboratories (A-D) provide microbiology service to seven public hospitals, including a university medical center, four other major medical centers, and two rehabilitation centers. Together they served a population of approximately 3 million in the Hong Kong Island (south and west), Kowloon (central), and the New Territory (south and north) regions of Hong Kong.

All strains were nonduplicate isolates obtained from consecutive clinical samples of hospitalized patients during the second half of 1998. Of 143 isolates obtained, three became nonviable during storage. The numbers of isolates from laboratories A, B, C, and D were 46, 39, 12, and 43, respectively.

MICs for penicillin and erythromycin were determined by the E-test method (AB Biodisk, Solna, Sweden), according to the manufacturer's instructions. MICs for ciprofloxacin, levofloxacin, and trovafloxacin were determined by a standardized broth microdilution procedure with cation-adjusted Mueller-Hinton broth supplemented with 2.5% lysed horse blood (3). All MIC results were interpreted according to National Committee for Clinical Laboratory Standards. For ciprofloxacin, an MIC value of $\geq$4 µg/mL was regarded as resistant (4). The isolates were serotyped by the quellung reaction (5) with sera of various reactivities (pools A to I, P to T, and selected major groups and serum factors) from the Statens Seruminstitut (Copenhagen, Denmark). All 15 ciprofloxacin-resistant isolates were compared with 16 ciprofloxacin-susceptible isolates, *S. pneumoniae* ATCC 49619, and the well-defined Spanish clones of serotypes 23F and 6B (SP264 ATCC 700669 and GM17 ATCC 700670, respectively), by PFGE after digestion of the genomic DNA with *Sma*I and *Apa*I, respectively (6).

Ciprofloxacin-susceptible isolates of serotypes 6A, 19F, and 23F, matching the serotypes in the ciprofloxacin-resistant isolates, were chosen for PFGE analysis. The 16 ciprofloxacin-susceptible isolates included the remaining 4 isolates of serotype 6A and 6 each of serotypes 19F and 23F, chosen randomly. Of the 16 ciprofloxacin-susceptible isolates, 5 were penicillin sensitive, 3 were intermediate, and 8 were resis-

Address for correspondence: PakLeung Ho, Department of Microbiology, Queen Mary Hospital, University of Hong Kong, Pokfulam Road, Pokfulam, Hong Kong SAR, China; fax: 852-2855-1241; e-mail: plho@hkucc.hku.hk tant. These ciprofloxacin-susceptible isolates were obtained from all four laboratories (A, 5; B, 4; C, 3; and D, 4). The Fisher exact or chi-square test was used for statistical analysis, with a value of <0.05 indicating statistical significance.

Of the 140 isolates, 18 (12.9%) and 87 (62.1%) were intermediate (MIC 0.11 µg/mL) and resistant (MIC ≥2 µg/mL) to penicillin, respectively. One hundred twelve (80%) of 140 were nonsusceptible to erythromycin (MIC ≥0.5 mg/mL). Fifteen of the 140 isolates were resistant to ciprofloxacin (four isolates from laboratory A, one from laboratory B, two from laboratory C, and eight from laboratory D). The 15 isolates had ciprofloxacin MICs of 4 µg/mL (4/15), 8 mg/mL (2/15), 16 µg/mL (5/15), and 32 µg/mL (4/15). All nine isolates with ciprofloxacin MICs 16 to 32 µg/mL and one with a ciprofloxacin MIC 8 µg/mL were intermediately resistant (2/10) or resistant (8/10) to levofloxacin. One and three isolates with ciprofloxacin MICs of 16 and 32 mg/mL, respectively, were intermediately resistant (1/4) or resistant (3/4) to trovafloxacin. All four trovafloxacin-nonsusceptible isolates were resistant to levofloxacin. All ciprofloxacin-resistant strains were from adults 54 to 88 years of age and were resistant to both penicillin and erythromycin. No isolates were obtained from the children in the 6- to 17-year age group. Penicillin-nonsusceptible strains were common in all other age groups: <2 years (9/11), 2 to 5 years (21/23), 18 to 49 years (9/11), 50 to 64 years (10/11), and ≥65 years (56/84).

Eighteen serotypes or serogroups were identified among the 123 typeable strains (Table). The most common serotypes were 23F (41.4%), 19F (18.6%), and 6B (9.3%). The combined proportions of these three serotypes by age group were as follows: <2 years (9/ 11), 2 to 5 years (20/23), 18 to 49 years (9/11), 50 to 64 years (9/11), and ≥65 years (50/84). Penicillin-nonsusceptible strains were more likely than susceptible ones to belong to these three serotypes (93 of 105 vs. 4 of 35, p<0.001). For the penicillin sensitive, intermediate, and resistant isolates, 10, 4, and 3 isolates were untypeable, respectively. Exclusive of the untypeable isolates, 7 (28%) of 25, 13 (92.9%) of 14, and 84 (100%) of 84 of the penicillin-sensitive, intermediate, and resistant isolates, respectively, were restricted to serogroups 6, 19, and 23. On the basis of identical serotypes, coverage of the recently licensed heptavalent-conjugated vaccine by age groups was 81.8% for those <2 years, 91.3% for 2 to 5 years, 81.8% for 18 to 49 years, 81.8% for 50 to 64 years, and 59.5% for ≥65 years.

PFGE analysis identified seven groups of DNA patterns when either *Sma*I or *Apa*I was used (Figure). The six subtypes of group A (A0-A5) were either identical (A0) to that of the Spanish 23F clone or differed from it by 1 to 4 bands (A1 to A5). Group A patterns were shared by 14 of 15 ciprofloxacin-nonsusceptible (all A1) and 12 of 16 ciprofloxacin-susceptible (3 A0, 3 A1, 3 A2, 1 A3, 1 A4, and 1 A5) isolates. The remaining five distinct profiles (B, C, D, E, F) were identified in each of the remaining five isolates. All strains with group A subtypes were either identical or closely related by *Apa*I analysis.

### Conclusions

PFGE analysis showed that most ciprofloxacin-resistant and ciprofloxacin-susceptible *S. pneumoniae* isolates were either identical or closely related to the Spanish 23F clone. This clone is dominant in Hong Kong, where it accounted for approximately 70% of all penicillin-resistant *S. pneumoniae*

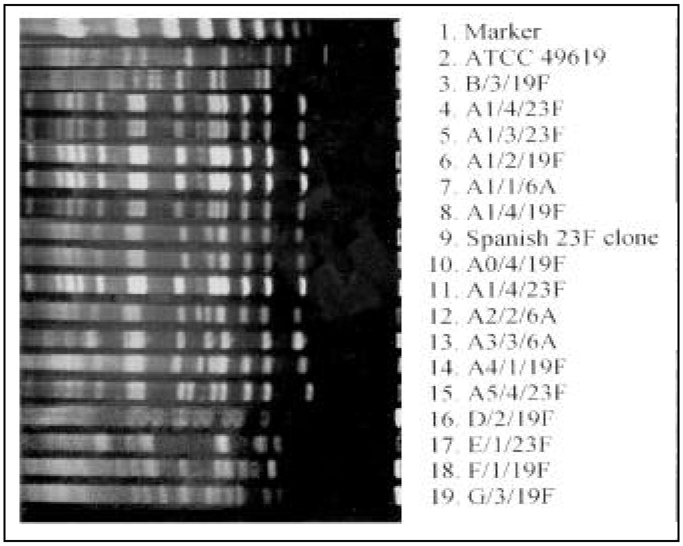

Figure. Pulsed-field gel electrophoresis (PFGE) patterns of *Sma*I-digested genomic DNA of *Streptococcus pneumoniae* isolates. Seven distinct patterns, including A and its subtypes (A0 to A5) and B to G, are shown. Labels indicate the PFGE pattern, hospital source, and serotype. Lane 1: molecular weight markers; lane 2: *S. pneumoniae* ATCC 49619; lanes 3 to 8: ciprofloxacin-resistant isolates; lane 9: Spanish 23F clone (ATCC 700669); lanes 10-19: ciprofloxacin-sensitive isolates.

isolates from 1994 to 1997 (7). Our data suggest that this clone has acquired fluoroquinolone resistance and is already widespread in Hong Kong. In contrast, in Canada and Spain fluoroquinolone-resistant pneumococci are also emerging, but with genetically diverse strains (4). Nonetheless, our finding raises the possibility that this fluoroquinolone-resistant variant of the Spanish 23F clone might spread internationally, as its ancestor did in the past decade (8). Therefore, infection control guidelines should be formulated for screening and isolating patients with fluoroquinolone-resistant *S. pneumoniae*. Furthermore, molecular analysis of fluoroquinolone-resistant strains from other countries compared with those from Hong Kong is also indicated to determine whether this clone has already disseminated outside Hong Kong.

Our data show that the Hong Kong fluoroquinolone-resistant clone is resistant to multiple other antibiotics, including penicillin, erythromycin, and clindamycin (2). This finding implies that the fluoroquinolone-resistant variant could be selected not only by fluoroquinolones but also by other antibiotics. In Sweden, for instance, cotrimoxazole has been shown to select for penicillin resistance in children attending day-care centers (9). Given the wide range of antibiotic classes involved, further emergence of the fluoroquinolone-resistant clone is likely. One potentially sinister situation would be dissemination of the clone to children. Although fluoroquinolone is rarely used in children in Hong Kong, the fluoroquinolone-resistant strain could spread, for example, through household contacts from adults to children. Its spread could readily be facilitated by the frequent misuse of antibiotics for upper respiratory tract infections in children. The therapeutic half-life of fluoroquinolones as treatment for pneumococci is now being challenged. Finally, three different serotypes (6A, 19F, 23F) were expressed by our strains that were closely related in the PFGE analysis. This is likely a result of capsular switching, as reported previously (10).

Table. Distribution of capsular types in 140 strains of mucosal *Streptococcus pneumoniae* with respect to age and resistance to ciprofloxacin

| Type | Age group (years) | | | | | Ciprofloxacin MIC ≥4 µg/mL | | |
|---|---|---|---|---|---|---|---|---|
| | <2 | 2-5 | 18-49 | 50-64 | ≥65 | No[a] (n=125) No. (%) | Yes (n=15) No. (%) | No. (%) |
| 19F | 4 | 3 | 1 | 4 | 14 | 19 (15.2) | 7 (46.7) | 26 (18.6) |
| 23F | 4 | 12 | 8 | 4 | 30 | 51 (40.8) | 7 (46.7) | 58 (41.4) |
| 6A | 1 | 1 | | | 3 | 4 (0.8) | 1 (6.7) | 5 (3.6) |

[a]Number of strains for other serotype or serogroup by age groups: <2 years (1 for 6B and 1 untypeable), 2 to 5 years (5 for 6B, 1 for 9V, and 1 untypeable), 18 to 49 years (1 for 10B and 1 for 35), 50 to 64 years (1 for 6B, 1 for 19C, and 1 for 34), and ≥65 years (6 for 6B, 1 for 7F, 1 for 9A/L, 1 for 10C/F, 1 for 11A, 3 for 13, 1 for 15B, 1 for 19B, 1 for 21, 1 for 22A, 5 for 35, and 15 untypeable). No isolates were obtained from the children in the 6- to 17-year age group. All strains with serotypes or serogroups other than 6A, 19F, and 23F were sensitive to ciprofloxacin.

In conclusion, most of the drug-resistant pneumococci in Hong Kong were of serotypes 6A/B, 19F, and 23F. Fluoroquinolone-resistant strains, which were found only in older adults, were genetically highly similar and probably have arisen by acquisition of fluoroquinolone resistance by the locally dominant Spanish 23F clone.

### Acknowledgments

We thank K.P. Klugman for providing the Spanish clones of serotypes 23F and 6B and K.H. Chow and K.H. Tsang for their technical support.

This work was supported by a grant from the University Research Committee/Committee on Research and Conference Grants, University of Hong Kong.

Dr. Ho is associate professor in the Department of Microbiology, University of Hong Kong. His interests include infectious diseases, epidemiology, and mechanisms of emerging antimicrobial resistance.

### References

1. Bartlett JG, Breiman RF, Mandell LA, File-TM J. Community-acquired pneumonia in adults: guidelines for management. The Infectious Diseases Society of America. Clin Infect Dis 1998;26:811-38.
2. Ho PL, Que TL, Tsang DN, Ng TK, Chow KH, Seto WH. Emergence of fluoroquinolone resistance among multiply resistant strains of *Streptococcus pneumoniae* in Hong Kong. Antimicrob Agents Chemother 1999;43:1310-3.
3. National Committee for Clinical Laboratory Standards. Methods for dilution antimicrobial susceptibility tests for bacteria that grow aerobically; approved standard. 5th ed. Villanova (PA): The Committee; 2000.
4. Chen DK, McGeer A, de Azavedo JC, Low DE. Decreased susceptibility of *Streptococcus pneumoniae* to fluoroquinolones in Canada. Canadian bacterial surveillance network. N Engl J Med 1999;341:233-9.
5. Austrian R. The quellung reaction, a neglected microbiologic technique. Mt Sinai J Med 1976;43:699-709.
6. Lefevre JC, Faucon G, Sicard AM, Gasc AM. DNA fingerprinting of *Streptococcus pneumoniae* strains by pulsed-field gel electrophoresis. J Clin Microbiol 1993;31:2724-8.
7. Ip M, Lyon DJ, Yung RW, Chan C, Cheng AF. Evidence of clonal dissemination of multidrug-resistant *Streptococcus pneumoniae* in Hong Kong. J Clin Microbiol 1999;37:2834-9.
8. Munoz R, Coffey TJ, Daniels M, Dowson CG, Laible G, Casal J, et al. Intercontinental spread of a multiresistant clone of serotype 23F *Streptococcus pneumoniae*. J Infect Dis 1991;164:302-6.
9. Melander E, Molstad S, Persson K, Hansson HB, Soderstrom M, Ekdahl K. Previous antibiotic consumption and other risk factors for carriage of penicillin-resistant *Streptococcus pneumoniae* in children. Eur J Clin Microbiol Infect Dis 1998;17:834-8.
10. Gherardi G, Inostrozo JS, O'Ryan M, Prado V, Prieto S, Arellano C, et al. Genotypic survey of recent beta-lactam-resistant pneumococcal nasopharyngeal isolates from asymptomatic children in Chile. J Clin Microbiol 1999;37:3725-30.
